# Correction: A review on advancements in carbon quantum dots and their application in photovoltaics

**DOI:** 10.1039/d2ra90018a

**Published:** 2022-02-23

**Authors:** Pawan Kumar, Shweta Dua, Ravinder Kaur, Mahesh Kumar, Geeta Bhatt

**Affiliations:** Department of Electronic Science, South Campus University of Delhi New Delhi-110021 India; Bhaskarcaharya College of Applied Sciences, University of Delhi New Delhi-110075 India; Deen Dayal Upadhyaya College, University of Delhi New Delhi-110075 India; CSIR-National Physical Laboratory (NPL) New Delhi-110012 India; Non-Collegiate Women’s Education Board, University of Delhi New Delhi-110007 India geeta.bhatt@bcas.du.ac.in

## Abstract

Correction for ‘A review on advancements in carbon quantum dots and their application in photovoltaics’ by Pawan Kumar *et al.*, *RSC Adv.*, 2022, **12**, 4714–4759, DOI: 10.1039/D1RA08452F

The authors regret that the affiliations were incorrectly shown in the original manuscript. The corrected list of affiliations is as shown above.

The Royal Society of Chemistry apologises for these errors and any consequent inconvenience to authors and readers.

## Supplementary Material

